# *In Vitro* Activity of Cefiderocol Compared to Other Antimicrobials against a Collection of Metallo-Beta-Lactamase-Producing Gram-Negative Bacilli from Southern Spain

**DOI:** 10.1128/spectrum.04936-22

**Published:** 2023-05-30

**Authors:** M. Delgado-Valverde, I. Portillo-Calderón, E. Recacha, P. Pérez-Palacios, A. Pascual

**Affiliations:** a Unidad Clínica de Enfermedades Infecciosas y Microbiología, Hospital Universitario Virgen Macarena, Sevilla, Spain; b Instituto de Biomedicina de Sevilla, Hospital Universitario Virgen Macarena/CSIC/Universidad de Sevilla, Sevilla, Spain; c Centro de Investigación Biomédica en Red en Enfermedades Infecciosas (CIBERINFEC), Instituto de Salud Carlos III, Madrid, Spain; d Departamento de Microbiología, Universidad de Sevilla, Sevilla, Spain; Weill Cornell Medicine

**Keywords:** cefiderocol, Gram-negative, *in vitro* activity, metallo-beta-lactamase

## Abstract

In this study, we aimed to comparatively evaluate the *in vitro* activity of cefiderocol versus other antimicrobials against a well-characterized collection of metallo-beta-lactamase (MBL)-producing Gram-negative bacilli (MBL-GNB) isolates from hospitals in Andalusia, Spain. We recovered 232 MBL-GNB from Andalusian hospitals, including 160 Enterobacterales and 72 nonfermenting Gram-negative bacilli belonging to 44 different clones (2015 to 2020). Cefiderocol and comparator MICs were determined with commercial methods (UMIC [Bruker] and EUMDROXF [Sensititre; Thermo Fisher], respectively). EUCAST breakpoints were used for all antimicrobials tested, and CLSI also was used for cefiderocol. Control strains used were E. coli ATCC 25922 and Pseudomonas aeruginosa ATCC 27853. Cefiderocol showed potent *in vitro* activity against isolates tested, regardless of breakpoint (susceptibility rates, 85.3% for EUCAST versus 96.6% for CLSI, *P < *0.001). MIC ranges for Enterobacterales and nonfermenting Gram-negative bacilli (NF-GNB) were ≤0.03 to 1 mg/L and 0.06 to 2 (IMP), 0.06 to 8 mg/L and 0.06 to 16 (VIM), 0.25 to 16 mg/L and 2 to 16 mg/L (NDM), respectively, and 0.25 to 8 mg/L for double MBL-producing Enterobacterales. By species, all cefiderocol-susceptible rates were over 90%, except Klebsiella oxytoca, Enterobacter cloacae, Escherichia coli, and Acinetobacter spp. Significant differences were observed comparing resistant isolates between Enterobacterales and NF-GNB by EUCAST (19.4% versus 4.2%, *P < *0.01), but not by CLSI (4.4% versus 1.4%, *P = *0.2). Cefiderocol was the most active antimicrobial tested. Cefiderocol showed excellent *in vitro* activity against MBL-GNB, especially NF-GNB; almost all isolates resistant to comparators were susceptible.

**IMPORTANCE** This article demonstrates the efficacy of cefiderocol against a large collection of well-characterized metallo-beta-lactamase-producing isolates, some of them even producing double carbapenemases. Furthermore, cefiderocol activity is compared to other novel broad-spectrum antimicrobials with activity against carbapenemases.

## INTRODUCTION

In recent decades, there has been a steady increase in infections caused by multidrug-resistant bacteria, with significant impact on morbidity, mortality, and health care costs (1–3).

The natural adaptive mechanisms of bacteria, together with the selective pressure of antimicrobial use, have allowed bacteria to develop multiple mechanisms of resistance to the different antimicrobial molecules available. One of the strategies to combat multidrug resistance is to develop new antimicrobials faster and more efficiently. The World Health Organization established a list of critical pathogens for which these new molecules should be designed as a priority, including Acinetobacter baumannii, Pseudomonas aeruginosa, and carbapenem-resistant Enterobacterales (4).

Carbapenemases are one of the main resistance mechanisms to carbapenems in Gram-negative bacilli. Metallo-beta-lactamases (MBLs) belong to Ambler class B; they hydrolyze nearly all beta-lactams except monobactams and are not inhibited by clavulanic acid and related inhibitors. Furthermore, MBLs are usually carried on plasmids that encode other resistance determinants, such as extended-spectrum beta-lactamases (ESBLs) or fluoroquinolone- and aminoglycoside-resistant genes, resulting in multiresistance profiles for which there are hardly any therapeutic options (5, 6).

Since 2017, a limited number of antimicrobials have been developed, only five of which have activity against carbapenemase producers, and only three of these are active against MBLs, including plazomicin (aminoglycoside), eravacycline (tetracycline), and cefiderocol (beta-lactam) (4). Poor activity against this type of carbapenemase has been observed with other new beta-lactam antimicrobial combinations, such as meropenem-vaborbactam, imipenem-relebactam, ceftazidime-avibactam, and ceftolozane-tazobactam. Consequently, the options for treating infections caused by MBL-producing bacteria are very limited (7).

Cefiderocol is the first approved siderophore cephalosporin whose activity is based on its iron-chelating property. This property allows cefiderocol to make use of the constitutive iron transport system of bacteria, in the manner of a Trojan horse, in order to penetrate the external membrane. Once in the periplasmic space, it binds to penicillin-binding protein 3, preventing peptidoglycan synthesis (8). This molecule was developed to target carbapenem-resistant isolates, demonstrating stability against the different classes of beta-lactamase (Ambler classes A to D), as well as strains with mutations in porins or efflux pumps. Despite its broad-spectrum activity against Gram-negative bacteria, it is not active against Gram-positive or anaerobic bacteria (9, 10).

Our group evaluated the activity of cefiderocol and comparators against a collection of high-risk clones of ESBL- and/or carbapenemase-producing Enterobacterales, A. baumannii, P. aeruginosa, and Stenotrophomonas maltophilia, and showed that it exhibited potent activity against the majority of isolates (11).

Those results were similar to those observed in other studies against multidrug-resistant isolates of these bacterial species (12, 13). Nevertheless, there are studies that have found a specific association between the presence or overexpression of certain beta-lactamases, such as some class A extended-spectrum beta-lactamases (PER, BEL, and SHV), class D (OXA-427), or MBLs (NDM, IMP, and SPM-1), and reduced cefiderocol activity in Gram-negative bacilli (14–18). In addition, some studies have linked the presence of mutations in TonB-dependent siderophore receptors to increased resistance to cefiderocol in some Enterobacterales species (19, 20).

The aim of this study was to comparatively evaluate the *in vitro* activity of cefiderocol and other available antimicrobials against a well-defined collection of recently isolated MBL-producing Gram-negative bacilli (MBL-GNB) from southern Spain.

## RESULTS

### Cefiderocol activity against MBL-GNB producers.

Overall, 85.3% and 96.6% of isolates were classified as susceptible using EUCAST and CLSI breakpoints, respectively. Significant differences in cefiderocol resistance rates were observed depending on whether EUCAST or CLSI criteria were used (*P* < 0.001). According to EUCAST, resistant isolates were 2 Klebsiella pneumoniae NDM-7 producers, 16 Klebsiella oxytoca VIM-1 producers, 2 E. coli NDM producers (1 NDM-1 and 1 NDM-5), 10 Enterobacter cloacae (1 NDM-1, 8 VIM-1, and 1 coproducing IMP-8 and OXA-48), 1 Citrobacter freundii VIM-1 producer, and 3 A. baumannii NDM-1 producers. Of the resistant isolates, 70.6% were ESBL coproducing.

MIC ranges, MIC_50_ and MIC_90_ values, and resistance and susceptibility percentages using EUCAST criteria are shown in Table 1. Enterobacterales and nonfermenting Gram-negative bacilli (NF-GNB) showed similar cefiderocol MIC ranges (≤0.03 to 16 mg/L and 0.06 to 16 mg/L, respectively) and the same MIC_90_ and MIC_50_ values, which were 4 mg/L and 1 mg/L, respectively.

**TABLE 1:** MIC ranges, MIC_50_ and MIC_90_ values, and resistance and susceptibility percentages for MBL-producing Enterobacterales and NF-GNB using EUCAST breakpoints^*h*^

Species or antibiotic	MIC range (mg/L)	MIC_50_ (mg/L)	MIC_90_ (mg/L)	Clinical category (%)		
				S	I	R
Enterobacterales (*n* = 160)						
Cefiderocol	≤0.03 to 16	1	4	80.6	NA	19.4
Piperacillin-tazobactam	8 to >32	>32	>32	0.6	NA	99.4
Cefepime	8 to >16	>16	>16	0	0	100
Ceftazidime-avibactam	16 to >16	>16	>16	0.6	NA	99.4
Ceftolozane-tazobactam	>8	>8	>8	0	NA	100
Aztreonam	≤1 to >32	>32	>32	9.4	0.6	90
Imipenem	2 to >8	8	>8	11.9	34.4	53.8
Imipenem-relebactam	2 to >8	4	>8	13.8	NA	86.3
Meropenem	0.5 to >16	16	16	20	30	50
Meropenem-vaborbactam	0.25 to >16	8	>16	56.3	NA	43.8
Amikacin	≤2 to >32	4	>32	88.8	NA	11.3
Tobramycin	0.5 to >4	>4	>4	6.9	NA	93.1
Colistin	≤0.5 to >16	≤0.5	≤0.5	95	NA	5
Tigecycline^*a*^	≤0.5 to >1	≤0.5	>1	65.6	NA	34.4
Fosfomycin^*b*^	≤16 to >64	32	>64	61.3	NA	38.8
Eravacycline^*c*^	0.125 to >0.5	0.25	>0.5	69.4	NA	30.6
NF-GNB (*n* = 72)						
Cefiderocol^*d*^	0.06 to 16	1	4	95.8	NA	4.2
Piperacillin-tazobactam^*e*^	8 to >32	>32	>32	0	1.4	93.1
Cefepime^*e*^	>16	>16	>16	0	0	94.4
Ceftazidime-avibactam^*e*^	>16	>16	>16	0	NA	94.4
Ceftolozane-tazobactam^*e*^	>8	>8	>8	0	NA	94.4
Aztreonam^*e*^	4 to >32	8	32	0	76.4	18.1
Imipenem	4 to >8	>8	>8	0	1.4	98.6
Imipenem-relebactam	4 to >8	>8	>8	0	NA	100
Meropenem	4 to >16	>16	>16	0	2.8	97.2
Meropenem-vaborbactam^*f*^	2 to >16	>16	>16	2.8	NA	91.7
Amikacin	≤2 to >32	8	>32	61.1	NA	38.9
Tobramycin	≤0.5 to >4	>4	>4	2.8	NA	97.2
Colistin	≤0.5 to 2	1	1	100	NA	0
Tigecycline^*g*^	≤0.5 to >1	>1	>1	NA	NA	NA
Fosfomycin^*g*^	32 to >64	>64	>64	NA	NA	NA
Eravacycline^*g*^	≤0.03 to >0.5	>0.5	>0.5	NA	NA	NA

a E. coli and Citrobacter koseri breakpoints.

b Intravenous breakpoints.

c E. coli breakpoints.

d PK/PD breakpoints (non-species related) for Acinetobacter spp.

e No EUCAST breakpoint for Acinetobacter spp.

f The beta-lactamases produced by the microorganisms either do not modify the parent carbapenem or are not affected by the inhibitor. Therefore, the addition of the beta-lactamase inhibitor does not add clinical benefit (41).

g No EUCAST breakpoint for Acinetobacter spp. and Pseudomonas spp.

h Isolates were classified as susceptible (S), susceptible, increased exposure (I), and resistant (R). NA, not applicable.

When EUCAST breakpoints were applied, significant differences were detected in the percentages of resistance between the two groups of isolates: Enterobacterales had 19.4% resistance, and NF-GNB had 4.2% (*P < *0.01), but not when using CLSI, where Enterobacterales showed 4.4%, and NF-GNB showed 1.4% (*P = *0.2) of resistant isolates. The resistance percentage was higher in both groups of isolates when the EUCAST breakpoint was applied. Overall categorical agreement (CA) between the two criteria for Enterobacterales was 85% and 97.2% for NF-GNB.

An analysis by MBL subtype is shown in Table 2. When EUCAST criteria were applied, VIM-producing isolates showed lower susceptibility than other MBLs; in contrast, when CLSI was used, almost all VIM-producing isolates were susceptible (76.9% versus 97.2%). All IMP-producing isolates were susceptible regardless of the clinical breakpoint used. Differences in susceptibility rates were observed for VIM and NDM subtypes. Lower cefiderocol activity was detected in the group of VIM-1- and NDM-1-producing isolates (72.8% and 73.7%, respectively, according to EUCAST breakpoints).

**TABLE 2:** MIC ranges, MIC_50_ and MIC_90_ values, resistance and susceptibility rates by MBL group (IMP, VIM or NDM) and subtypes for cefiderocol

MBL group or producer (no.)	MIC range (mg/L)	MIC_50_ (mg/L)	MIC_90_ (mg/L)	EUCAST clinical category (%)^*a*^		CLSI clinical category (%)^*a*^		
				S	R	S	I	R
IMP group (55)	≤0.03 to 2	0.25	1	100	0	100	0	0
IMP-8 (29)	≤0.03 to 2	0.25	1	100	0	100	0	0
IMP-16 (15)	0.125 to 1	0.25	1	100	0	100	0	0
IMP-22 (1)	NC*	NC	NC	100	0	100	0	0
IMP-23 (10)	0.06 to 1	1	2	100	0	100	0	0
VIM group (108)	0.06 to 8	2	4	76.9	23.1	97.2	2.8	0
VIM-1 (92)	0.06 to 8	2	4	72.8	27.2	96.7	3.3	0
VIM-2 (16)	0.125 to 2	0.5	1	100	0	100	0	0
NDM group (57)	0.25 to 16	1	4	86	14	93	3.5	3.5
NDM-1 (19)	0.25 to 16	2	16	73.7	26.3	84.2	5.3	10.5
NDM-5 (7)	0.5 to 8	NC	NC	85.7	14.3	85.7	14.3	0
NDM-7 (31)	0.5 to 4	1	2	93.5	6.5	100	0	0

a When EUCAST breakpoints were used, the clinical categories used to classify isolates were susceptible (S) and resistant (R). When CLSI breakpoints were used, the clinical categories used to classify isolates were susceptible (S), intermediate (I), and resistant (R). NC, not calculated because number of microorganisms was less than 10; NC*, not calculated because number of microorganisms was less than 2.

Cefiderocol MIC values for the control strains, E. coli ATCC 25922 and P. aeruginosa ATCC 27853, were within the EUCAST guidelines.

### Activity of cefiderocol and comparators against MBL-producing Enterobacterales.

Cefiderocol, amikacin, and colistin were the most active antimicrobials (>80% susceptibility rates). Eravacycline and meropenem-vaborbactam showed moderate activity against this collection (69.4% and 56.3% susceptibility rates, respectively), and imipenem-relebactam was not active (only 13.8% of susceptible isolates). At least 90% of isolates were categorized as resistant to aztreonam, cefepime, ceftazidime-avibactam, ceftolozane-tazobactam, piperacillin-tazobactam, and tobramycin.

MIC ranges, MIC_50_ and MIC_90_ values, and resistance and susceptibility percentages for each species are shown in Table 3. The cefiderocol MIC_90_ varied according to species, with values between 2 mg/L and 8 mg/L. This parameter could not be calculated for E. coli due to the small sample size in the collection (*n* < 10). Cefiderocol was the most active agent against K. pneumoniae and showed excellent activity against C. freundii (>90% susceptible isolates). K. oxytoca, E. cloacae, and E. coli showed lower susceptibility rates (55.6%, 69.7%, and 75%, respectively) when EUCAST breakpoints were used. Using CLSI breakpoints, however, K. oxytoca and E. cloacae susceptibility rates increased to 97.2% and 87.9%, respectively. There were no differences in colistin activity between species. Meropenem-vaborbactam activity was influenced by bacterial species, with susceptibility rates ranging between 27.5% and 93.9%.

**TABLE 3:** MIC ranges, MIC_50_ and MIC_90_ values, and resistance and susceptibility rates by species for cefiderocol and commercial comparators in Enterobacterales

Species or antibiotic	MIC range (mg/L)	MIC_50_ (mg/L)	MIC_90_ (mg/L)	Clinical category (%)^*d*^		
				S	I	R
K. pneumoniae (*n* = 69)						
Cefiderocol	0.06 to 4	1	2	97.1	NA	2.9
Piperacillin-tazobactam	8 to >32	>32	>32	1.4	NA	98.6
Cefepime	16 to >16	>16	>16	0	0	100
Ceftazidime-avibactam	>16	>16	>16	0	NA	100
Ceftolozane-tazobactam	>8	>8	>8	0	NA	100
Aztreonam	≤1 to >32	64	64	4.3	0	95.7
Imipenem	2 to >8	>8	>8	5.8	14.5	79.7
Imipenem-relebactam	2 to >8	>8	>8	7.2	0	92.8
Meropenem	1 to >16	>16	>16	11.6	11.6	76.8
Meropenem-vaborbactam	0.5 to >16	>16	>16	27.5	NA	72.5
Amikacin	≤2 to >32	4	>32	79.7	NA	20.3
Tobramycin	2 to >4	>4	>4	5.8	NA	94.2
Colistin	≤0.5 to >16	≤0.5	≤0.5	92.8	NA	7.2
Tigecycline^*a*^	≤0.5 to >1	≤0.5	2	49.3	NA	50.7
Fosfomycin^*b*^	≤16 to >64	32	>64	66.7	NA	33.3
Eravacycline^*c*^	0.125 to >0.5	0.5	>0.5	58.0	NA	42
K. oxytoca (*n* = 36)						
Cefiderocol	0.25 to 8	2	4	55.6	NA	44.4
Piperacillin-tazobactam	>32	>32	>32	0	NA	100
Cefepime	8 to >16	>16	>16	0	0	100
Ceftazidime-avibactam	>16	>16	>16	0	NA	100
Ceftolozane-tazobactam	>8	>8	>8	0	NA	100
Aztreonam	≤1 to >32	>32	>32	13.9	0	86.1
Imipenem	2 to >8	4	>8	8.3	44.4	47.2
Imipenem-relebactam	2 to >8	4	>8	8.3	0	91.7
Meropenem	0.5 to >16	8	16	8.3	50.0	41.7
Meropenem-vaborbactam	0.25 to >16	8	16	72.2	NA	27.8
Amikacin	≤2 to 8	4	8	100	NA	0
Tobramycin	2 to >4	>4	>4	2.8	NA	97.2
Colistin	≤0.5 to 2	≤0.5	≤0.5	100	NA	0
Tigecycline^*a*^	≤0.5 to >1	≤0.5	1	72.2	NA	27.8
Fosfomycin^*b*^	≤16 to >64	32	>64	55.6	NA	44.4
Eravacycline^*c*^	0.125 to >0.5	0.5	>0.5	77.8	NA	22.2
E. cloacae (*n* = 33)						
Cefiderocol	0.5 to 8	2	8	69.7	NA	30.3
Piperacillin-tazobactam	32 to >32	>32	>32	0	NA	100
Cefepime	8 to >16	>16	>16	0	0	100
Ceftazidime-avibactam	>16	>16	>16	0	NA	100
Ceftolozane-tazobactam	>8	>8	>8	0	NA	100
Aztreonam	≤1 to >32	>32	>32	12.1	0	87.9
Imipenem	2 to >8	4	8	12.1	66.7	21.2
Imipenem-relebactam	2 to >8	4	8	15.2	NA	84.8
Meropenem	0.5 to >16	4	16	42.4	51.5	6.1
Meropenem-vaborbactam	0.5 to >16	2	8	93.9	NA	6.1
Amikacin	≤2 to 8	≤2	4	100	NA	0
Tobramycin	2 to >4	4	>4	6.1	NA	93.9
Colistin	≤0.5 to >16	≤0.5	≤0.5	93.9	NA	6.1
Tigecycline^*a*^	≤0.5 to >1	≤0.5	1	78.8	NA	21.2
Fosfomycin^*b*^	≤16 to >64	32	>64	48.5	NA	51.5
Eravacycline^*c*^	0.125 to >0.5	0.25	>0.5	72.7	NA	27.3
C. freundii (*n* = 14)						
Cefiderocol	≤0.03 to 4	1	2	92.9	NA	7.1
Piperacillin-tazobactam	>32	>32	>32	0	NA	100
Cefepime	8 to >16	>16	>16	0	0	100
Ceftazidime-avibactam	0.5 to >16	>16	>16	7.1	NA	92.9
Ceftolozane-tazobactam	>8	>8	>8	0	NA	100
Aztreonam	≤1 to >32	16	>32	7.1	7.1	85.7
Imipenem	2 to >8	2	8	50	28.6	21.4
Imipenem-relebactam	2 to >8	2	8	50	NA	50
Meropenem	0.5 to 16	4	32	42.9	28.6	28.6
Meropenem-vaborbactam	0.25 to >16	4	16	78.6	NA	21.4
Amikacin	≤2 to 32	≤2	32	85.7	NA	14.3
Tobramycin	4 to >4	>4	>4	0	NA	100
Colistin	≤0.5 to 1	≤0.5	≤0.5	100	NA	0
Tigecycline^*a*^	≤0.5 to 1	≤0.5	1	78.6	NA	21.4
Fosfomycin^*b*^	≤16 to 32	≤16	>64	78.6	NA	21.4
Eravacycline^*c*^	0.125 to >0.5	0.25	>0.5	78.6	NA	21.4
E. coli (*n* = 8)						
Cefiderocol	0.06 to 16	NC	NC	75	NA	25
Piperacillin-tazobactam	>32	NC	NC	0	NA	100
Cefepime	>16	NC	NC	0	0	100
Ceftazidime-avibactam	>16	NC	NC	0	NA	100
Ceftolozane-tazobactam	>8	NC	NC	0	NA	100
Aztreonam	≤1 to >32	NC	NC	25	0	75
Imipenem	2 to >8	NC	NC	12.5	37.5	50
Imipenem-relebactam	2 to >8	NC	NC	25	NA	75
Meropenem	0.5 to >16	NC	NC	12.5	12.5	75
Meropenem-vaborbactam	0.5 to >16	NC	NC	37.5	NA	62.5
Amikacin	≤2 to >32	NC	NC	75	NA	25
Tobramycin	≤0.5 to >4	NC	NC	50	NA	50
Colistin	≤0.5	NC	NC	100	NA	0
Tigecycline^*a*^	≤0.5	NC	NC	100	NA	0
Fosfomycin^*b*^	≤16 to >64	NC	NC	62.5	NA	37.5
Eravacycline^*c*^	0.125 to 0.5	NC	NC	100	NA	0

a E. coli and C. koseri breakpoints.

b Intravenous breakpoints.

c E. coli breakpoints.

d EUCAST breakpoints were used, and isolates were classified in clinical categories of susceptible (S), susceptible, increased exposure (I), and resistant (R). NC, not calculated because the number of microorganisms was less than 10; NA, not applicable.

The activity of cefiderocol and comparators was also assessed in terms of MBL group. MIC ranges, MIC_50_ and MIC_90_ values, and clinical categories according to EUCAST and/or CLSI criteria are shown in Table 4. MIC ranges were ≤0.03 mg/L to 1 mg/L (IMP group), 0.06 mg/L to 8 mg/L (VIM group), 0.25 mg/L to 16 mg/L (NDM group), and 0.25 mg/L to 8 mg/L (double-CBP producers); however, all MBL groups had MIC_50_ values below ≤2 mg/L. All IMP-producing isolates were susceptible to cefiderocol irrespective of the breakpoints used, and lower activity was observed in the VIM group (70.6% by EUCAST, 96.5% by CLSI). Regardless of the breakpoint used, NDM production or double carbapenemases did not appear to influence cefiderocol activity, and susceptibility rates remained above 90%.

**TABLE 4:** MIC ranges, MIC_50_ and MIC_90_ values, and resistance and susceptibility percentages by MBL group (IMP group, VIM group, NDM group, and double-carbapenemase group) for cefiderocol in Enterobacterales and NF-GNB^*a*^

MBL group producer (subtype)	MIC range (mg/L)	MIC_50_ (mg/L)	MIC_90_ (mg/L)	EUCAST clinical category (%)^*a*^			CLSI clinical category (%)^*a*^		
				S	I	R	S	I	R
Enterobacterales									
IMP group (8, 22)	≤0.03 to 1	0.5	1	100	NA	0	100	0	0
VIM group (1, 4)	0.06 to 8	2	4	70.6	NA	29.4	96.5	3.5	0
NDM group (1, 5, 7)	0.25 to 16	1	2	90.6	NA	9.4	94.3	3.8	1.9
Double carbapenemases	0.25 to 8	1	2	91.7	NA	8.3	91.7	8.3	0
NF-GNB									
IMP group (8, 16, 23)	0.06 to 2	0.25	1	100	NA	0	100	0	0
VIM group (1, 2)	0.125 to 2	0.5	2	100	NA	0	100	0	0
NDM-1	2 to 16	NC	NC	25	NA	75	75	0	25

a When EUCAST breakpoints were used, the clinical categories used to classify isolates were susceptible (S), susceptible, increased exposure (I), and resistant (R). When CLSI breakpoints were used, isolates were classified as susceptible (S), intermediate (I), and resistant (R). NC, not calculated because number of microorganisms was less than 10; NA, not applicable.

### Activity of cefiderocol and comparators against MBL-producing NF-GNB.

In this group of microorganisms, cefiderocol and colistin were again the most active agents, with 95.8% and 100%, respectively, of susceptible microorganisms when EUCAST breakpoints were used. Aztreonam showed moderate activity (76.4%) against this group of isolates, although in the susceptible, increased exposure category (Table 1). Cefiderocol activity increased to 98.6% when CLSI criteria were applied. None of the comparators showed activity in this collection, except for amikacin, with moderate susceptibility (61.1%).

The activity of cefiderocol and comparators according to species is given in Table 5. No differences in cefiderocol activity were detected among the included species: all Pseudomonas spp. and 25% of Acinetobacter spp. were susceptible when EUCAST breakpoints were followed. When CLSI criteria were applied, 75% of Acinetobacter spp. were susceptible.

**TABLE 5:** MIC ranges, MIC_50_ and MIC_90_ values, and resistance and susceptibility percentages by species for cefiderocol and commercial comparators in NF-GNB^*d*^

Species or antibiotic	MIC range (mg/L)	MIC_50_ (mg/L)	MIC_90_ (mg/L)	Clinical category (%)		
				S	I	R
P. aeruginosa (*n* = 60)						
Cefiderocol	0.06 to 2	0.25	1	100	NA	0
Piperacillin-tazobactam	8 to >32	>32	>32	0	1.67	98.3
Cefepime	>16	>16	>16	0	0	100
Ceftazidime-avibactam	>16	>16	>16	0	NA	100
Ceftolozane-tazobactam	>8	>8	>8	0	NA	100
Aztreonam	4 to >32	8	16	0	90	10
Imipenem	4 to >8	>8	>8	0	1.7	98.3
Imipenem/relebactam	4 to >8	>8	>8	0	NA	100
Meropenem	4 to >16	>16	>16	0	3.3	96.7
Meropenem-vaborbactam	2 to >16	>16	>16	0	3.3	96.7
Amikacin	≤2 to >32	16	>32	53.3	NA	46.7
Tobramycin	4 to >4	>4	>4	0	NA	100
Colistin	≤0.5 to 2	1	1	100	NA	0
Tigecycline^*a*^	≤0.5 to >1	>1	>1	NA	NA	NA
Fosfomycin^*a*^	32 to >64	>64	>64	NA	NA	NA
Eravacycline^*a*^	0.25 to >0.5	>0.5	>0.5	NA	NA	NA
P. putida (*n* = 8)						
Cefiderocol	0.125 to 2	NC	NC	100	NA	0
Piperacillin-tazobactam	>32	NC	NC	0	0	100
Cefepime	>16	NC	NC	0	0	100
Ceftazidime-avibactam	>16	NC	NC	0	NA	100
Ceftolozane-tazobactam	>8	NC	NC	0	NA	100
Aztreonam	16 to >32	NC	NC	0	12.5	87.5
Imipenem	>8	NC	NC	0	0	100
Imipenem-relebactam	>8	NC	NC	0	NA	100
Meropenem	>16	NC	NC	0	0	100
Meropenem-vaborbactam	>16	NC	NC	0	NA	100
Amikacin	≤2	NC	NC	100	NA	0
Tobramycin	≤0.5 to >4	NC	NC	12.5	NA	87.5
Colistin	≤0.5 to 1	NC	NC	100	NA	0
Tigecycline^*a*^	>1	NC	NC	NA	NA	NA
Fosfomycin^*a*^	64 to >64	NC	NC	NA	NA	NA
Eravacycline^*a*^	>0.5	NC	NC	NA	NA	NA
Acinetobacter spp. (*n* = 4)						
Cefiderocol^*b*^	2 to 16	NC	NC	25	NA	75
Piperacillin-tazobactam^*a*^	>32	NC	NC	NA	NA	NA
Cefepime^*a*^	>16	NC	NC	NA	NA	NA
Ceftazidime-avibactam^*a*^	>16	NC	NC	NA	NA	NA
Ceftolozane-tazobactam^*a*^	>8	NC	NC	NA	NA	NA
Aztreonam^*a*^	16 to >32	NC	NC	NA	NA	NA
Imipenem	>8	NC	NC	0	0	100
Imipenem-relebactam	>8	NC	NC	0	NA	100
Meropenem	>16	NC	NC	0	0	100
Meropenem-vaborbactam^*c*^	>16	NC	NC	NA	NA	NA
Amikacin	≤2-8	NC	NC	100	NA	0
Tobramycin	≤0.5 to >4	NC	NC	25	NA	75
Colistin	≤0.5 to 1	NC	NC	100	NA	0
Tigecycline^*a*^	≤0.5	NC	NC	NA	NA	NA
Fosfomycin^*a*^	64 to >64	NC	NC	NA	NA	NA
Eravacycline^*a*^	0.03 to 0.06	NC	NC	NA	NA	NA

a No EUCAST breakpoint.

b PK/PD breakpoints (non-species related).

c The beta-lactamases produced by the microorganisms either do not modify the parent carbapenem or are not affected by the inhibitor. Therefore, the addition of the beta-lactamase inhibitor does not add clinical benefit.

d EUCAST breakpoints were used, and isolates classified as susceptible (S), susceptible, increased exposure (I), and resistant (R). NA, not applicable (<10 microorganisms).

MIC ranges, MIC_50_ and MIC_90_ values, and clinical categories of NF-GNB by the MBL group using CLSI and EUCAST breakpoints are shown in Table 4. MIC ranges were 0.06 mg/L to 2 mg/L (IMP group), 0.125 mg/L to 2 mg/L (VIM group), and 2 mg/L to 16 mg/L (NDM group). One hundred percent of IMP and VIM group isolates were susceptible, irrespective of the breakpoint used. Cefiderocol showed very little activity against NDM group isolates relative to IMP and VIM producers: 75% of isolates were resistant when EUCAST criteria were followed and 25% when the CLSI breakpoint was applied; however, only four isolates were included.

## DISCUSSION

In this study, we analyzed a well-characterized collection of more than 200 MBL-GNB recently isolated from different hospitals in Andalusia, and we detected excellent activity of cefiderocol compared to other antimicrobials. These findings corroborate those observed in previous studies, which demonstrated excellent activity of the same antimicrobial against carbapenem-resistant GNB (21, 22).

Cefiderocol was the most active beta-lactam antimicrobial against our collection of MBL-producing isolates. In general, in the Enterobacterales group, better activity was detected, with the older antimicrobials with less favorable pharmacokinetic/pharmacodynamic (PK/PD) properties, such as amikacin and colistin, which are used as second-line drugs in the therapeutic arsenal. Amikacin and colistin were the only antimicrobials whose activity surpassed that of cefiderocol. These results were similar to those obtained in the NF-GNB group, although aztreonam showed higher activity in this group, and tigecycline was excluded from the analysis, as EUCAST breakpoints are not established.

Some studies evaluating the cefiderocol activity against GNB lack a detailed characterization of the underlying resistance determinants in their bacterial collections and do not take into account the presence of enzymatic resistance mechanisms that somehow infer resistance to antimicrobials of the beta-lactam family, such as cefiderocol. While this mechanism has not been reliably identified as a cause of resistance to cefiderocol, several studies have shown an association between the presence of certain carbapenemase types, such as MBLs, and decreased susceptibility to this antimicrobial (14, 15, 17); in particular, increased copy number and expression of NDM-5 have been associated with resistance to cefiderocol (18). However, in our study, no decrease in cefiderocol activity was detected in NDM-5-producing Enterobacterales isolates relative to isolates producing other NDM subtypes, such as NDM-1.

In connection with this, previous studies have compared the activity of cefiderocol against MBL-producing isolates (15, 16, 21–23). Mushtaq et al. analyzed a collection of carbapenemase-producing GNBs, including 209 MBL-producing isolates with more than 90 NDM producers, and observed resistance percentages of 37% for Enterobacterales and 21% for NF-GNB, using EUCAST criteria. Furthermore, they found variable cefiderocol activity depending on the MBL type, showing reduced cefiderocol activity, with rates below 50% in isolates harboring the NDM type, but above 80% when the MBL was of the VIM or IMP type (16). These results are not entirely consistent with those observed in our study. We observed differences in susceptibility rates (using EUCAST breakpoints) according to type of MBL producer, although the percentage of cefiderocol-resistant isolates detected in our collection of MBL-producing isolates was lower (19.4% in Enterobacterales and 4.2% for NF-GNB). Additionally, in our collection, VIM-producing isolates, in particular, VIM-1 producers, showed a reduced susceptibility profile to cefiderocol compared with NDM-producing isolates, regardless of subtype, which does not agree with Mushtaq et al. results (16). It should be noted, however, that the number of NDM-producing isolates was also lower. The influence of the breakpoints used on the activity of cefiderocol is noteworthy. In general, the susceptibility percentages were higher when CLSI breakpoints were applied, regardless of the type of MBL. Resistant isolates were detected only in the NDM group, 1 E. coli isolate and 1 A. baumannii isolate, both NDM-1 producers.

Focusing specifically on the results for the Enterobacterales group, a decrease in susceptibility rates was observed among K. oxytoca, E. cloacae, and E. coli isolates. All K. oxytoca isolates were VIM-1 producers, and less than 60% were susceptible to cefiderocol. E. cloacae and E. coli produced a high proportion of VIM-1 or NDM (80% and 100%, respectively). The increase in cefiderocol MIC associated with MBL-producing E. cloacae and E. coli has been described in previous research by our group and others (11, 15, 24). Our results for K. oxytoca, on the other hand, do not agree with those observed in previous studies. Longshaw et al. found that 92% of K. oxytoca isolates not susceptible to carbapenems were susceptible to cefiderocol, although they did not specify the mechanism of carbapenem resistance in these isolates, and the number of isolates was lower than in our collection (25). This phenomenon was not detected in the other Enterobacterales species in our analysis. Cefiderocol was active against the vast majority of isolates of the K. pneumoniae group, in which the proportion of VIM-1 and NDM carbapenemases was greater than 90%, and against C. freundii isolates, which also harbored a high proportion of VIM-1 (85.7%). Indeed, in a very recent study testing the activity of cefiderocol in a collection of more than 100 carbapenem-resistant enterobacterial isolates, 6.7% of which were VIM-1 or NDM-1 producers, all were susceptible to cefiderocol (26).

On the other hand, when the NF-GNB group was analyzed in detail, cefiderocol showed decreased activity against NDM-producing isolates, which were harbored exclusively in the Acinetobacter species group and absent from the Pseudomonas species group of isolates. As for VIM-producing isolates, unlike the Enterobacterales group, all Pseudomonas spp. were susceptible to cefiderocol. Previous studies have shown variable activity of cefiderocol against MBL-producing NF-GNB isolates (16, 23, 24, 27). To our knowledge, this is the first study to evaluate the *in vitro* activity of cefiderocol against Pseudomonas putida isolates, although the collection of VIM-producing isolates evaluated was small.

In our study, significant differences were detected in the percentage of susceptible isolates in the Enterobacterales group versus the NF-GNB group when EUCAST breakpoints were applied. However, when CLSI breakpoints were followed, no significant differences were detected; in the Enterobacterales group, the percentage of resistance to cefiderocol decreased from 19.4% to 4.4%, while in the NF-GNB group, it remained stable. This difference could be explained by the higher number of VIM-1- and NDM-producing isolates in the Enterobacterales group. Since not all VIM-1- or NDM-producing isolates are resistant to cefiderocol, this could rule out a possible direct relationship between cefiderocol resistance and the presence of both MBL genes, suggesting that other factors related to the regulation of expression of these genes or iron uptake could be involved, as well as factors arising from species-specific characteristics (9).

Our results show that the proportion of resistant isolates in the two groups differed significantly, depending on the criteria applied to interpret antimicrobial susceptibility. The lack of harmonization between different committees with respect to cefiderocol breakpoints can lead to discrepant results (28). This difference in criteria particularly affected the clinical category in our collection of Enterobacterales isolates since most of them had MIC values on the borderline of susceptibility and resistance (4 mg/L), depending on the committee used. These differences were also observed by Mushtaq et al. in a collection of Enterobacterales, P. aeruginosa, and A. baumannii isolates (16).

Our study has some strengths: first, the use of previously validated commercial methods with control strains allowed us to study a large number of isolates in a simple and easily applicable way as part of the daily routine of the clinical microbiology laboratory. We compared a large number of new-generation beta-lactam antimicrobials with activity against carbapenemases, such as ceftazidime-avibactam, ceftolozane-tazobactam, imipenem-relebactam, and meropenem-vaborbactam, and the results showed the superior activity of cefiderocol against both Enterobacterales and NF-GNB. Second, this study evaluated isolates producing more than one carbapenemase, sometimes two MBLs, although no influence on cefiderocol activity was detected in this group. Finally, we analyzed the impact of the subtype of each MBL group on cefiderocol activity.

One of the limitations of our study is the low representation of certain MBL-producing bacterial species, such as E. coli, A. baumannii, and P. putida, against which this antimicrobial appears to have lower activity. It would be worthwhile, therefore, to conduct a study with a larger collection of isolates of these species with a wider representation of MBL producers and nonproducers.

In conclusion, cefiderocol remains a good therapeutic option against MBL-producing GNB isolates, especially VIM-producing NF-GNB, such as P. aeruginosa and P. putida, and against Enterobacterales, including NDM-producing isolates. However, previous studies have observed that the presence of NDM could facilitate the development of resistance to this antimicrobial in some bacterial species, so in these cases, the use of cefiderocol should be monitored (29). Further studies are needed to elucidate the underlying mechanisms of resistance, as are well-designed PK/PD studies and clinical trials to clarify and standardize the appropriate clinical breakpoints.

## MATERIALS AND METHODS

### Bacteria.

A total of 232 MBL-GNB isolates were recovered from Andalusian hospitals in Spain over a 5-year period (2015 to 2020), including 160 Enterobacterales and 72 nonfermenting Gram-negative bacilli (NF-GNB) (Table 6). Forty-four different clones were selected from a well-characterized collection from the reference laboratory of the Andalusian program for the surveillance and control of health care-associated infections and antibiotic stewardship (PIRASOA), based at the Hospital Universitario Virgen Macarena, Seville, Spain (30, 31). Twelve of the isolates (5.2%) were double-carbapenemase producers, and 116 Enterobacterales (72.5%) also harbored an extended-spectrum beta-lactamase (ESBL). Bacterial species and corresponding beta-lactam resistance determinants (type of carbapenemase and ESBL) are shown in Table 6.

**TABLE 6:** Distribution and number of isolates for each type of resistant determinant (MBL ± ESBL) tested

Microorganism (*n* = 232)	Clone or resistance determinant	No. of isolates
Enterobacterales (*n* = 160)		
K. pneumoniae (*n* = 69)	NDM-7 + CTX-M-15	29
	NDM-1 + CTX-M-15	12
	NDM-5 + CTX-M-15	5
	VIM-1 + CTX-M-15	15
	IMP-8 + CTX-M-15	2
	IMP-8	3
	VIM-1 + OXA-48 + CTX-M-15	2
	IMP-8 + OXA-48 + CTX-M-15	1
K. oxytoca (*n* = 36)	VIM-1 + CTX-M-15	10
	VIM-1 + CTX-M-15 + CMY-2	10
	VIM-1	15
	VIM-1 + OXA-48	1
E. cloacae (*n* = 33)	VIM-1 + SHV-12	22
	VIM-1 + SHV-12 + CTX-M-9	2
	VIM-1 + OXA-48	1
	VIM-1 + IMP-8 + SHV-12	1
	IMP-8 + OXA-48	2
	IMP-8	3
	NDM-1	1
	IMP-22	1
C. freundii (*n* = 14)	VIM-1	5
	VIM-1 + SHV-12	4
	VIM-1 + OXA-48	2
	VIM-1 + OXA-245 + CTX-M-9 + SHV-12	1
	VIM-4 + NDM-1	1
	IMP-8	1
E. coli (*n* = 8)	VIM-1	2
	NDM-7	2
	NDM-1	2
	NDM-5	2
NF-GNB (*n* = 72)		
P. aeruginosa (*n* = 60)	IMP-8	20
	IMP-16	15
	IMP-23	10
	VIM-2	15
P. putida (*n* = 8)	VIM-1	7
	VIM-2	1
Acinetobacter spp. (*n* = 4)	NDM-1	4

### Bacterial and resistance gene identification.

Isolate identification was confirmed at the reference laboratory by matrix-assisted laser desorption ionization–time of flight mass spectrometry (MALDI-TOF MS; MALDI-TOF Biotyper 3.1; Bruker microFlex; Madrid, Spain). Until September 2018, characterization of ESBLs (CTX-M, SHV, and TEM group) and carbapenemases (NDM, VIM, KPC, IMP, and OXA-48 groups) in Enterobacterales, P. aeruginosa, and P. putida, as well as of oxacillinases in Acinetobacter spp., was performed by PCR and DNA sequencing (32–34).

As of October 2018, resistance determinants were characterized by whole-genome sequencing (WGS) through in-house MiSeq sequencing (Illumina, San Diego, CA, USA). Libraries were prepared with the Nextera XT DNA sample preparation kit (Illumina San Diego, CA, USA) and then sequenced using Illumina MiSeq (300-bp paired-end reads) sequencing technology. CLC Genomics Workbench software (Qiagen, Netherlands) was used for *de novo* assembly of Illumina reads, ensuring at least 30× average coverage. Genomes were analyzed in the resistance database at https://www.genomicepidemiology.org (ResFinder 4.0) (35–37).

The collection of isolates for this study was performed based on clonal relationship analysis by pulsed-field gel electrophoresis (PFGE). PFGE analysis of XbaI (Enterobacterales)-, SpeI (P. aeruginosa and P. putida)-, and ApaI (Acinetobacter spp.)-digested DNA (http://www.cdc.gov/pulsenet) was used to determine the degree of genetic relatedness between isolates. A dendrogram was created with Fingerprinting 3.0 software (Bio-Rad), using the Dice coefficient with position tolerance settings of 1% optimization and 1.2% band position tolerance. Isolates differing by two or more bands in PFGE assays were assigned to a different pulsotype; only one isolate for pulsotype was selected for analysis.

### Drug susceptibility testing.

*In vitro* activity of cefiderocol (0.03 to 32 mg/L) was performed by broth microdilution with UMIC cefiderocol (Bruker) and the Sensititre EUMDROXF panel (Thermo Fisher) as comparators, according to the manufacturers’ instructions. Escherichia coli ATCC 25922 and P. aeruginosa ATCC 27853 were used as control strains.

EUCAST criteria were applied to all antimicrobials from the commercial panel. For cefiderocol, both EUCAST (≤2 mg/L for susceptible and >2 mg/L for resistant) and CLSI breakpoints (≤4 mg/L for susceptible, 8 mg/L for intermediate, and ≥16 mg/L for resistant) were applied. When following EUCAST criteria, cefiderocol PK/PD breakpoints were used for Acinetobacter species isolates, and E. coli breakpoints were applied for tigecycline and eravacycline for Enterobacterales. Categorical agreement (CA) between the two committees was calculated for cefiderocol (38). EUCAST guidelines for quality control of antimicrobials were followed with the control strains (39, 40).

### Statistical analysis.

Significant differences between the results obtained in the susceptibility tests were analyzed by Pearson’s χ^2^.

### Data availability.

The genomes were published in the ENA database at https://www.ebi.ac.uk/ena/browser/home under accession no. PRJEB53686.
